# Reimagining Anatomy Education: The Potential of Virtual Learning

**DOI:** 10.7759/cureus.96534

**Published:** 2025-11-10

**Authors:** Jerome Linkwinstar, Imran Ashraf, Kaynat Noor Chaudhry, Geniya Jery, Nadeem Saleem, Husham Akhlaq

**Affiliations:** 1 Orthopaedic Surgery, St. Sofia General Hospital, Sofia, BGR; 2 Anatomy, Imperial College London, London, GBR; 3 Anatomy, Sofia Medical University, Sofia, BGR; 4 Anatomy, Plovdiv Medical University, Plovdiv, BGR

**Keywords:** anatomy, blending learning approach, covid-19, online teaching & learning, virtual sessions

## Abstract

Objective: This study aimed to determine whether a single live interactive online anatomy session could enhance student confidence, engagement and perceived effectiveness. Secondary objectives included assessing accessibility, availability and overall satisfaction.

Methods: A live upper limb anatomy revision session was delivered online to 45 medical students and graduates by a surgical trainee. Teaching included a structured lecture followed by an interactive discussion, with the recording made available for later review. Following the session, participants completed a questionnaire assessing accessibility, availability, educational effectiveness, engagement, and satisfaction. Quantitative items were measured using a 5-point Likert scale, and qualitative responses were thematically analysed to identify recurrent insights. Internal consistency was assessed using Cronbach’s alpha, with α ≥ 0.70 considered acceptable. Descriptive statistics were used for quantitative analysis.

Results: All 45 attendees completed the questionnaire. Of these, 36 (80%) were medical students, six (13%) were medical graduates, and three (7%) came from other backgrounds. Twenty-seven participants (60%) were female and 18 (40%) were male. Second and third-year medical students formed the largest group (51%). Accessibility was rated highly (mean = 4.47 ± 0.66, 95% CI 4.28-4.66; t(44) = 13.2, p < 0.001), with 96% reporting that online delivery was at least as accessible as institutional lectures. Technological barriers were minimal (mean = 2.13 ± 1.47; t(44) = -3.9, p < 0.001). Availability was improved, as 62% found the session was more accessible than traditional lectures and 67% reported that their schools did not routinely provide recorded anatomy lectures. Effectiveness was rated positively (mean = 3.9 ± 0.98, 95% CI 3.61-4.19; t(44) = 5.8, p < 0.001), and 60% agreed that self-directed learning remained useful for dissection preparation. Confidence increased significantly after the session. Engagement (mean = 4.09 ± 0.79, 95% CI 3.85-4.33; t(44) = 8.9, p < 0.001) and perceived benefits (mean = 4.24 ± 0.74, 95% CI 4.00-4.48; t(44) = 9.8) were significantly above neutrality (p < 0.001), with no scores below 3.

Conclusion: A single live, interactive online anatomy session significantly improved student confidence and perceived learning effectiveness while being rated as highly accessible and engaging. Online teaching offers flexibility and a wider reach, but it also has limitations, particularly the lack of practical dissection experience. Although this model cannot replicate the tactile experience of cadaveric teaching, it demonstrates a scalable, low-resource approach to broadening access to anatomy education. Future implementations could integrate short virtual modules into blended curricula to complement practical dissection and ensure equitable learning opportunities across diverse training environments.

## Introduction

The COVID-19 pandemic led to a sudden transition to online learning across many fields, including medical education, where universities rapidly adopted virtual and hybrid delivery models to ensure curricular continuity [1]. Anatomy, a core component of medical training, is typically taught through in-person lectures and dissections, but it faced significant challenges [2]. Traditionally, students learned anatomy by working directly with cadavers, gaining essential hands-on experience [3]. However, with social distancing and university closures, educators had to find virtual alternatives to maintain quality education [4].

While online anatomy education existed before the COVID-19 pandemic, its adoption in medical training accelerated during this period [5,6]. Virtual dissection tools, 3D anatomical models, and interactive platforms have since been integrated into curricula worldwide, demonstrating potential to enhance flexibility and accessibility [7,8]. Several studies have reported comparable short-term academic outcomes between online and traditional anatomy teaching, particularly when virtual resources are combined with interactive components [6,9]. Nonetheless, most existing research has focused on semester-long or multi-session modules, leaving a gap in understanding how brief, single-session interventions might impact student engagement, confidence, and perceived learning effectiveness.

In the post-pandemic era, as medical schools move toward blended curricula, there is increasing recognition that technology-based educational formats can complement traditional cadaveric learning [10]. They offer the advantages of flexibility, cost-effectiveness, and equitable access, particularly for students in geographically or resource-limited settings, while promoting active participation through interactive features [2,4,11]. However, challenges remain, including the lack of hands-on dissection, which is critical for developing practical anatomical knowledge and surgical skills [9,12,13]. Technical issues and fewer opportunities for collaborative learning also complicate the transition. While students value the convenience of online platforms, concerns persist about their impact on spatial awareness and preparedness for clinical practice [9,12,13].

Despite the rapid adoption of digital learning tools, there remains limited evidence on the educational value of short, single-session online anatomy teaching. This study was designed to address that gap by evaluating the educational effectiveness, accessibility, and perceived value of a single live, interactive online anatomy session. We hypothesized that even a single, well-structured virtual session could significantly enhance learners’ confidence, engagement, and perceived comprehension. By assessing both quantitative and qualitative outcomes, this study aims to clarify the role of short-format online teaching within modern anatomy education.​

## Materials and methods

Medipath International conducted this cross-sectional study by designing and delivering an online Upper Limb Anatomy revision session. The session aimed to provide accessible, comprehensive teaching to medical students and medical graduates pursuing further postgraduate clinical education, focusing on identifying and explaining the function of major nerves, vessels, and muscles. To maximise participation, the session was promoted through social media and peer recommendations. No exclusion criteria were applied.

The single session was delivered live by a surgical trainee and lasted about one hour. A total of 45 students, from various locations, participated. The teaching format included a structured lecture followed by an interactive discussion, during which students asked questions in real time. The recorded session was uploaded to the platform within 24 hours after the event for ongoing access.

After the teaching session, participants were invited to complete a questionnaire on the same online platform. The questionnaire evaluated accessibility, availability, and educational effectiveness. It included both quantitative and qualitative components. Quantitative items used a standard 5-point Likert scale (1 = strongly disagree, 2 = disagree, 3 = neutral, 4 = agree, 5 = strongly agree) [14]. Open-ended questions allowed detailed feedback. The questionnaire was reviewed by two independent medical educators for face validity and piloted with a small group of medical students (n = 5) to ensure clarity and content relevance. The internal consistency of the questionnaire was confirmed using Cronbach’s α = 0.82, indicating acceptable internal reliability [15]. Demographic data (level of education, gender, and country of residence) were also collected. The complete questionnaire is included in the Appendix.

Participation in the survey was voluntary. Submission of responses was considered as implied informed consent. The principles set out in the Declaration of Helsinki are not applicable to this project, as it constitutes a service evaluation focused on a single teaching session [16]. As this project was classified as an educational service evaluation rather than human subjects research, formal institutional review board (IRB) approval was not required. All data were anonymised under the General Data Protection Regulation (GDPR) and stored securely on the platform [17]. Only the Medipath International panel members had access to the anonymised data to maintain privacy and integrity.

Data analysis was performed using Microsoft Excel (Microsoft Corporation, Redmond, USA). Descriptive statistics (mean, median, standard deviation (SD), interquartile range (IQR), confidence intervals and p-value from one sample t-tests) summarised demographic characteristics and Likert-scale responses. Qualitative responses were analysed thematically following Braun and Clarke’s six-step framework. Two independent reviewers (a surgical trainee and a medical educator) performed initial coding, with discrepancies resolved through discussion until consensus was reached. Themes were refined to reflect recurring patterns in participants’ perceptions of accessibility, engagement, and overall experience. Triangulation and inter-rater agreement ensured analytical rigour and reduced bias. By combining quantitative and qualitative approaches, the study aimed to provide an understanding of participants’ experiences and perceptions of online anatomy teaching.

## Results

All participants (n = 45) completed a questionnaire assessing the accessibility, availability, and effectiveness of the teaching session (see the Appendix for the complete questionnaire). Of the cohort, 36 (80%) identified as medical students, 6 (13%) as postgraduate trainees, and 3 (7%) selected “other.” Female participants represented 27 (60%) of respondents, while male participants accounted for 18 (40%). The most commonly reported year of study was the second and third years of medical school, comprising 23 (51%) of the attendees.

Accessibility

Participants rated the online platform highly for accessibility (mean = 4.47± 0.66, 95% CI 4.28-4.66, median = 5.0, IQR = 1.0), significantly higher than the neutral midpoint of 3. Most respondents (96%, n = 43) agreed or strongly agreed that the Medipath International online series was as accessible or more accessible than their standard institutional lectures (mean = 3.91± 0.99, 95% CI 3.61-4.20; t(44) = 6.3, p < 0.001, median = 4.0, IQR = 2.0). 

Technological difficulties were less frequently reported (mean = 2.13 ± 1.47, 95% CI 1.70-2.56, median = 1.0, IQR = 2.0), although some students noted intermittent connectivity issues and occasional platform lag (Table 1). Qualitative feedback supports these findings, highlighting “easy to join”, “clear presentation”, and “minimal technical interruption” as major strengths.

**Table 1:** Accessibility Outcomes

Question	Mean± SD	Median	t(df)	IQR	P-value
Online anatomy session accessibility	4.47 ± 0.66	5	13.2 (44)	1.0	<0.001
Technological limitations (reverse coded, higher = more diff.)	2.13 ± 1.47	1	–3.9 (44)	2.0	<0.001
Online anatomy session more accessible than standard medical school lectures	3.91 ± 0.99	4.0	6.3 (44)	2.0	<0.001

Overall, the findings suggest that the virtual format provided a flexible, user-friendly learning environment with limited technical barriers.

Availability

Two-thirds of participants (67%, n = 30) reported that their medical school did not routinely provide recorded anatomy lectures. Among those who did, access was evenly divided between recordings available within 24 hours and those accessible later, although all were available 24/7. When asked to compare availability, 28 (62%) reported that the Medipath session was more readily accessible than traditional lectures, whereas 15 (33%) observed no difference. Overall ratings of availability were significantly above neutral (p < 0.001). The high accessibility and availability ratings reflect that on-demand access and flexible scheduling were valued features of the online approach.

​Effectiveness and confidence

The perceived educational effectiveness of the online format was rated positively (mean = 3.9 ± 0.98, 95% CI 3.61-4.19; t(44) = 5.8, p < 0.001). In addition, 27 (60%) agreed that textbooks and self-directed learning remained helpful in preparing for dissection sessions, though responses were more variable (mean = 3.7± 1.1).

**Figure 1:**
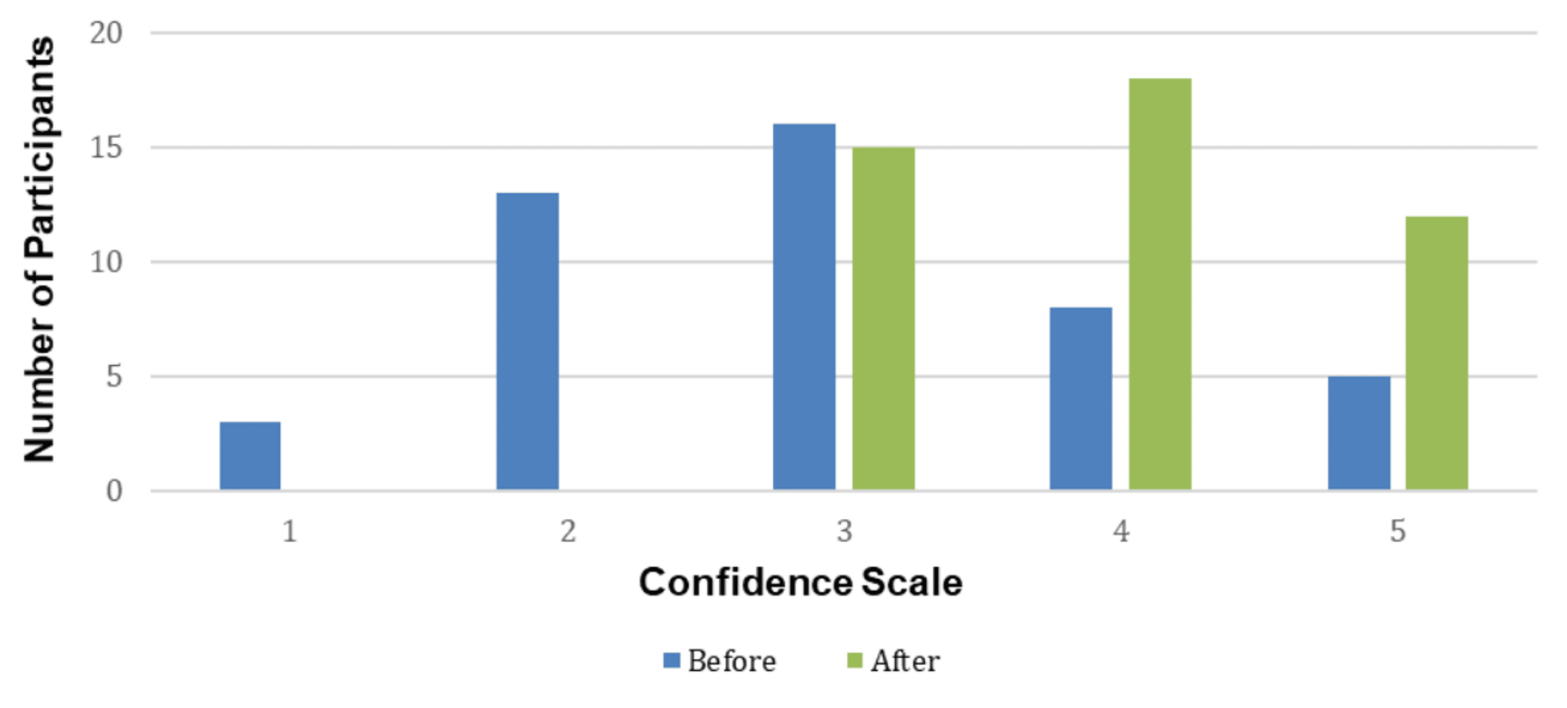
Comparison Between Pre- and Post Sessions in Confidence

A comparison of pre- and post-session confidence levels revealed a statistically significant improvement (mean difference = 0.83 ± 0.31, t(44) = 2.7, p = 0.009; 95% CI 0.21-1.45), as shown in Figure 1. Participants reported increased confidence in recalling anatomical relationships and understanding clinical correlations.

Qualitative comments supported this quantitative trend, with several respondents pointing out that the structured review “clarified nerve pathways”, “made anatomical structural relations easier” and “made previously confusing areas easier to remember.”

Engagement and perceived benefit

Engagement (mean = 4.09 ± 0.79, 95% CI 3.85-4.33; t(44) = 8.9, p < 0.001, median = 4.0, IQR = 2.0) and perceived benefit (mean = 4.24 ± 0.74, 95% CI 4.00-4.48; t(44) = 9.8, p < 0.001, median = 4, IQR = 1.0) both scored significantly above neutrality (Table 2). Importantly, no participants rated these domains below 3.

**Table 2:** Engagement and Perceived Benefit Scores

	Mean	Median	t(df)	IQR	p-value
Engagement	4.09 ± 0.79	4.0	8.9 (44)	2.0	<0.001
Perceived Benefit	4.24 ± 0.74	4.0	9.8 (44)	1.0	<0.001

A strong positive correlation was observed between engagement and perceived benefit (r = 0.81, p < 0.001), indicating that students who felt more engaged also perceived greater educational value (Table 2).

Qualitative analysis revealed three major themes: (1) interactivity enhances focus, (2) concise structure aids retention, and (3) visual resources reinforce spatial understanding. Representative comments included “the interactive Q&A was very good”, “excellent teaching" and “the diagrams made complex structures easier to follow.”

## Discussion

Effectiveness of online anatomy education

This study demonstrates that participation in a single live, interactive online anatomy session improved students’ self-reported confidence. A majority of participants (60%) reported that the online session was more effective than traditional lecture-based teaching, suggesting that well-designed digital sessions can positively influence learners’ perceived understanding. Unlike studies focusing mainly on asynchronous modules or pre-recorded content, this investigation emphasises the benefits of synchronous engagement, where students can ask questions in real time and clarify complex concepts [2,9]. By allowing students to interact in real time, the session likely enhanced confidence, illustrating that interactivity strengthens learning outcomes and that well-executed online anatomy sessions can rival traditional teaching in promoting understanding [6,18]. Despite lasting only one hour, the session had a measurable impact, demonstrating that well-designed, focused online interventions can be both practical and effective, particularly for learners with limited access to formal instruction or facing time constraints. These parallels strengthen the argument that well-designed online sessions can be as practical as conventional teaching in supporting knowledge acquisition.

While participants valued the accessibility and engagement of the session, qualitative feedback indicated that some participants desired greater depth and longer sessions, reflecting persistent challenges in replacing hands-on dissection. Previous studies similarly note that tactile experience is critical for developing spatial awareness and surgical skills [12,13]. Blended approaches help address these limitations: Darras et al. demonstrated that combining virtual dissection tools with online sessions enhances spatial awareness [19], while Rowe et al. and Bankar et al. highlighted the value of 3D models and virtual dissection tables in replicating aspects of hands-on training [11,20]. Evidence further suggests that integrating online lectures with laboratory-based sessions improves examination performance compared to online-only formats [21]. Overall, these findings reinforce that online anatomy teaching builds foundational knowledge and confidence, but its full potential is realised when combined with interactive, in-person experiences.

Accessibility and availability

The study showed that the anatomy session delivered via an online platform was perceived as more accessible and available than traditional lectures, with 62% reporting greater availability, highlighting the ability of virtual platforms to overcome geographic and institutional barriers. Prior studies have similarly highlighted accessibility as a significant strength of online education, particularly for students in remote or resource-constrained settings [2,22]. The ability to provide both live and recorded content further enhances flexibility, allowing learners to revisit material at their own pace, which is consistent with recent reports that asynchronous access contributes positively to student satisfaction and learning outcomes [6,23].

Despite these advantages, the study also identified challenges. Some participants experienced technological barriers, including unstable internet connectivity, which hindered the learning experience. Similar limitations have been reported in previous studies, with Rajab et al. noting that poor infrastructure can exacerbate disparities in learning opportunities [23]. Yoo et al. highlighted that, although online teaching is feasible, reliable platforms and institutional support are essential to maintain fair access [21]. This study found that two-thirds of respondents indicated that their medical schools did not provide an alternative platform for anatomy revision, suggesting that institutional provision of online resources remains inconsistent.

It should also be recognised that individual learning styles influence accessibility and satisfaction with online education. Each student brings unique preferences for how they engage with educational material, which in turn affects motivation and learning outcomes, particularly in the context of e-learning during the pandemic [24]. Senol et al. demonstrated that learning styles can significantly impact study duration and academic success, suggesting that the effectiveness of online resources varies across learners [25]. Barut et al. observed that study preferences vary by gender, academic year, and regional access to learning resources, suggesting that flexibility and institutional support are essential to meet diverse learner needs [26].

Overall, online platforms can improve the accessibility of anatomy education, but their effectiveness depends on reliable technology, institutional support, and accommodation of diverse learning styles. Providing robust digital resources, offline access, and adaptable teaching strategies is essential to ensure online learning is effective and equitable for all students.

Engagement & satisfaction

Participants reported high engagement and satisfaction with the online anatomy session, highlighting interactivity, clarity, and overall learning experience. These findings show that even a single, one-hour live lecture with interactive discussion can foster meaningful student involvement, particularly when real-time question-and-answer tools are available.

These results align with prior research demonstrating the impact of interactivity on engagement. Armstrong et al. found that incorporating interactive polling (via Mentimeter) into revision lectures significantly increased participation (30.1% vs. 6.4% for non-interactive questions) and improved test performance [27]. Similarly, a mixed-method study at Stellenbosch University showed that students valued clear communication, accessible course materials, and usable digital platforms, though the absence of physical interaction sometimes limited satisfaction [28].

In a cross-sectional study at Arabian Gulf University, students expressed a preference for face-to-face demonstrations for matters involving spatial orientation, complex anatomical structures, and clinical relevance, even though online formats were generally accepted for theoretical teaching [29]. The juxtaposition suggests that while students appreciate online anatomy teaching for its convenience and flexibility, aspects such as hands-on visualisation and spatial learning remain less satisfactory when delivered virtually only.

Studies also highlight the limitations of purely online formats for certain types of learning. At Arabian Gulf University, students preferred face-to-face demonstrations for spatial orientation, complex anatomical structures, and clinical relevance, despite generally accepting online formats for theoretical teaching [28]. The juxtaposition suggests that while students appreciate online anatomy teaching for its convenience and flexibility, aspects such as hands-on visualisation and spatial learning remain less satisfactory when delivered virtually only.

Research has explored which digital tools best support engagement in anatomy education. Adnan et al. indicated that virtual dissection tables, 3D anatomy models, and interactive platforms are highly rated; however, students’ preferences vary by academic level. More senior learners tend to favour tools that allow manipulation and detailed structure, while juniors often prefer guided digital resources with simpler interfaces [30]. Similarly, a study on eLearning in introductory anatomy and physiology showed engagement differed across subgroups depending on the relevance and usability of materials for assessment [31].

The findings of this study suggest that even brief, well-structured live online sessions can achieve high levels of student satisfaction and perceived effectiveness when they include meaningful interactivity and clear delivery. However, as others have observed, satisfaction does not always equate to improved performance. Pickering et al. reported that although students expressed strong satisfaction with technology-enhanced learning tools, such as screencasts, eBooks, and Massive Open Online Courses, these perceptions did not always correspond to higher engagement or better assessment results [32]. This highlights the need for online anatomy sessions that not only engage students but also lead to measurable learning gains.

Overall, satisfaction and engagement in online anatomy education are shaped by multiple factors, including the teaching modality (live versus asynchronous), the degree of interactivity (e.g., opportunities to ask questions or use audience response tools), the learner’s academic level, and the quality of digital materials. To maximise engagement, educators should focus on incorporating interactive elements, maintaining clear and well-paced instruction, and aligning content with learner needs-particularly when teaching complex or spatially oriented material.

Limitations

This study offers valuable insights into the role of online anatomy education, yet several limitations should be considered when interpreting the findings. The most significant limitation is the small sample size (n = 45), which restricts the statistical power and generalisability of the results. Although the sample was sufficient for preliminary analysis, a larger and more diverse cohort would allow for stronger inferences about the broader medical student population. Voluntary participation also introduces potential self-selection bias, as students with favourable attitudes toward digital learning or higher motivation levels may have been more likely to participate and report positive experiences. This bias may partly explain the high engagement and satisfaction scores observed.

Another key limitation lies in the reliance on self-reported outcomes to assess confidence, engagement, and perceived learning effectiveness. While subjective measures capture valuable learner perspectives, they do not necessarily reflect objective knowledge acquisition or long-term retention. This reliance limits the ability to draw firm conclusions about educational effectiveness. Incorporating objective assessments, such as pre- and post-session knowledge tests or follow-up evaluations, would help validate these findings and clarify whether reported confidence translates into measurable learning gains.

The absence of a control group also constrains interpretation. Without comparison to an equivalent cohort receiving in-person instruction, it remains uncertain whether improvements in confidence or engagement were attributable to the online format itself or to the educational content and delivery style. Future research should therefore adopt a randomised or quasi-experimental design, comparing online, blended, and traditional modalities to better isolate the effects of each. 

Technological barriers also presented challenges, as some participants experienced issues with internet connectivity and accessibility, which may have affected their learning experience. Future research should explore strategies to overcome these challenges, such as providing offline access to lecture materials.

Furthermore, while online platforms facilitate knowledge sharing, they lack the hands-on experience vital to anatomy education. Cadaveric dissection remains a core component of anatomical training, and the absence of tactile and spatial engagement is a significant limitation of virtual learning. Prior research suggests that a blended learning approach, combining online lectures with practical sessions, may offer a more comprehensive educational experience [24].

Lastly, the study focused on short-term outcomes, such as immediate post-session confidence and engagement, without assessing long-term knowledge retention or its influence on clinical practice. Future longitudinal studies should investigate whether the benefits of online anatomy education persist over time and translate into improved performance in practical and clinical settings. Such refinements would help establish the true educational value of online anatomy teaching and clarify its optimal role within modern, hybrid medical curricula.

## Conclusions

This study demonstrates that even a brief, well-structured live online anatomy session can enhance students’ confidence, engagement, and perceived understanding. Participants valued the accessibility and flexibility of the online format, which enabled learning across geographic and institutional boundaries. These findings suggest that short, interactive online sessions can serve as effective educational tools when thoughtfully designed and delivered in real time. In the context of post-pandemic medical education, such approaches offer a practical means of maintaining educational continuity while promoting equitable access to learning opportunities.

Nevertheless, the study also highlights the enduring importance of hands-on anatomical experience. Online teaching cannot fully replicate the spatial and tactile skills gained through cadaveric dissection, but it can complement traditional instruction within a blended curriculum. For medical educators, integrating concise, interactive online modules alongside in-person practical sessions may represent an optimal approach, one that combines accessibility with experiential depth. Future research should focus on scaling and standardising this hybrid model to evaluate its long-term educational impact and ensure sustainable improvements in anatomy training.

## Appendices

Accessibility 

1. I find the online platform for Medipath International's anatomy series readily accessible. (1 = strongly disagree, 5 = strongly agree)

2. I find it difficult to access the online platform to attend the Anatomy series due to technological limitations. (1 = strongly disagree, 5 = strongly agree)

3. In comparison to the standard format of medical school lectures, I find the Medipath International online anatomy lecture series more accessible. (1 = strongly disagree, 5 = strongly agree)

Availability

1. My medical school provides a platform to review recorded anatomy lectures. Yes/No

2. If Yes, which statement is accurate (regarding timing of availability): <24hrs or >24hrs

3. My medical school provides an alternative online resource for anatomy revision. Yes/No

4. I think that the Medipath International online anatomy series is more readily available than traditional lecture-based anatomy teaching delivered in medical school. (1 = strongly disagree, 5 = strongly agree) 

Efficacy

1. I find the online anatomy lecture series provided by Medipath International a more effective way of learning than traditional medical school lecture-based anatomy sessions. (1 = strongly disagree, 5 = strongly agree)

2. I find using anatomy textbooks/self-directed learning most effective in preparing for practical (prosection based/cadaveric dissection) sessions. (1 = strongly disagree, 5 = strongly agree)

Engagement & benefit

1. How confident were you in this topic before this training? (1 = very unconfident, 5 = very confident)

2. How confident were you in this topic after this training? (1 = very unconfident, 5 = very confident)

3. How engaging did you find the training? (1 = very disengaging, 5 = very engaging)

4. How helpful was the content? (1 = very unhelpful, 5 = very helpful)
